# Manufacturing data spaces applications in europe - A survey

**DOI:** 10.1016/j.dib.2025.112149

**Published:** 2025-10-15

**Authors:** Alexandros Nizamis, Panagiotis Gkonis, Dimosthenis Ioannidis, Aristotelis Ntafalias, Dimitrios Tzovaras, Panagiotis Trakadas

**Affiliations:** a Department of Digital Industry Technologies, National and Kapodistrian University of Athens, NKUA Euboea, Dirfies Messapies, 34400, Greece; b Information Technologies Institute, Center for Research and Technology Hellas, 6th km Charilaou-Thermi, Thessaloniki, 57001, Greece; c Motor Oil Hellas S.A, Marousi, Athens, 15124, Greece; d Department of Port Management and Shipping, National and Kapodistrian University of Athens, NKUA Euboea, Dirfies Messapies, 34400, Greece

**Keywords:** Data spaces, Manufacturing data spaces, Data space connectors, Data spaces review

## Abstract

Nowadays, Data Spaces concept and corresponding technologies are driving the implementations related to data sharing ecosystems in collaborative value chains, especially in Europe. One of the first domains that has adopted and promoted the Data Spaces concept is Manufacturing. In this work, a survey of applications based on the concept of Data Spaces in European Manufacturing is presented. The work provides to the readers a series of findings regarding application areas of Data Spaces, reference architectures, and data connectors used, along with insights regarding the development stage and the data used in each Data Space application for Manufacturing domain.

## 1 Introduction

In the 00s, the concept of Data Space was introduced [1] as an abstraction of data management systems to enable heterogeneous data integration. In the last decade, this concept of this concept has evolved significantly, transcending its original scope of data abstraction. It now encompasses critical aspects such as data sovereignty, interoperability, security, governance, and trust that are the key challenges in data sharing scenarios at the same time. To address these challenges, the Industrial Data Space initiative [2] was launched. This initiative in conjunction with the efforts [3] of the European Union (EU) has been instrumental in the formalization and advancement of the Data Spaces concept to further support the industry to address the growing need for secure, sovereign, and interoperable data sharing.

**Fig. 1:**
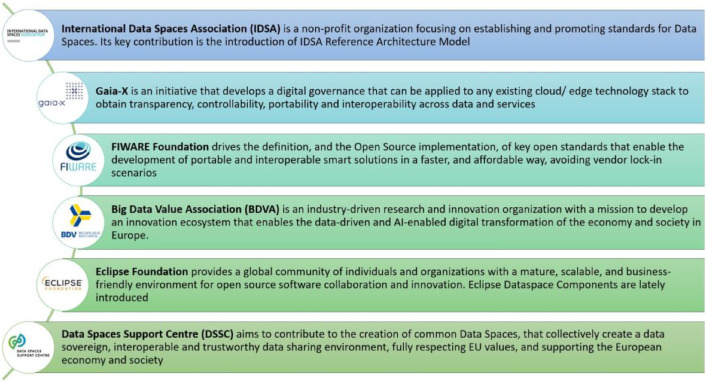
Initiatives that Drive Data Spaces Development in Europe

Today, the Data Spaces concept is a cornerstone of European research and innovation supported by numerous European Initiatives (Fig. 1). Notable among these are the International Data Spaces Association (IDSA) [4], FIWARE [5], Big Data Value Association (BDVA) [6], and Gaia-X [7]. They provide concepts, reference architectures, and technical components to enable the realization of use cases related to the Data Spaces. The IDSA Reference Architecture Model (RAM), has emerged as a pivotal framework for the implementation of Data Spaces in Europe. Moreover, Gaia-X contributed to the realization of Data Spaces, by providing a framework regarding cloud infrastructure. Lately, Eclipse Foundation [8] has joined these efforts by introducing open-source software components for Data Spaces.

Recognizing the strategic importance of Data Spaces, the European Commission (EC) funded the creation of a Data Spaces Support Center (DSSC) [9] to support the creation of data sharing ecosystems based on this paradigm. The goal of the EC data strategy [3] is the establishment of Common European Data Spaces [10] that will make data available for the benefit of European businesses and citizens.

Furthermore, the explosion of smart factories in the eras of Industry 4.0 and 5.0 (Table 1) promoted the adoption of connected machines, sensors, and AI systems. Data Spaces offer a robust framework to manufacturers for aggregating and securely sharing data across such cyber-physical environments. Moreover, Manufacturing has been designated by the EC as one of the 14 key sectors related to the development of Common European Data Spaces. Additionally, Digital Manufacturing Platforms [19] were a core subject of EC-funded research. They were focused on providing platforms and services for zero defect manufacturing [20], edge-to-cloud deployments [21], and sustainable manufacturing [22,23]. However, a shift from European Platforms to European Data Spaces was observed. To date, research projects have been funded, such as Data Space 4.0 [24] to promote Data Spaces in Manufacturing. EC-funded projects, UNDERPIN [25] and SM4RTENANCE [26] aim to enable a common and standardized way to build and maintain Manufacturing Data Spaces in Europe and to ensure access to European portfolio of Manufacturing Data Space assets.

**Table 1:** Related key concepts description.

Key concept	Description
Industry 4.0 and 5.0	Industry 4.0 concept is referred to the integration of digital technologies into manufacturing and industry, transforming traditional production processes and promoting automation. Industry 5.0 follows Industry 4.0 by adopting its findings but also focusing in value generation and human-machines collaboration. Its core concepts are human-centricity, sustainability and trustworthiness [11].
Manufacturing and Smart Manufacturing	Manufacturing is defined [12] as the entirety of interrelated economic, technological, and organizational measures directly connected with the processing/machining of materials, i.e., all activities directly contributing to the making of goods. Smart Manufacturing [13] uses Industries 4.0/5.0 concepts and technologies to create a connected and intelligent production ecosystem. It is considered as a successor of traditional Manufacturing. *It should be noted that in current study we are using the term Manufacturing but we are referring to Smart Manufacturing following the terminology of EC that uses the term Data Spaces for Manufacturing [14]
Data Space	Data Space is an interoperable framework, based on common governance principles, standards, practices and enabling services, for trusted data transactions between participants [15].
Data Connectors	Data (Space) connector is a technical component that is run by (or on behalf of) a data space participant and provides participant agent services, with similar components run by (or on behalf of) other participants. A connector can provide more functionality than is strictly related to connectivity. The connector offers technical modules that implement data interoperability functions, authentication etc. [16]
Metadata Brokers and Catalogues	These are components in a Data Space that keep registry of metadata related to data connectors such as self-descriptions of a connector including information about data available, issuer, issue date, authorization tokens etc. so to promote data discovery [16]. They are registries of information about the offered interfaces to access a data source, the owner of the source and the metadata of the offered data [17].
Data Provider and Data Consumer	Two are the main roles in data sharing scenarios based on Data Spaces concept: The Data Provider that is an entity providing data to the ecosystem and the Data Consumer, that is an entity consuming data from the ecosystem [18]

However, the state-of-the-art related to Data Spaces applications for Manufacturing domain has not yet been analysed. Therefore, the current landscape of Data Spaces related to Manufacturing, and the relevant applications are studied. To the best of our knowledge, no prior work has collected and analyzed studies focused on the application of Data Spaces in Manufacturing domain. The findings of the analysis shed light on the implementation and adoption of Data Spaces in Manufacturing, from various perspectives. In particular, the current study answers questions related to the areas of application of Data Spaces in Manufacturing and their development stage, the reference architectures and data connector implementations used. Moreover, it provides information on the data and data models available in the Data Spaces applications for Manufacturing. It should be noted that the current study is focused on Europe as the concept was developed mainly there with the support of the EC.

Even though Data Spaces have started to gain popularity in other regions, such as Asia [27] and South America [28], they are still in the early stages. Therefore, the current study focuses on Europe, where the higher number of applications enables the extraction of useful insights related to the above-mentioned questions.

A summary of Industrial Data Spaces is presented [29] along with a presentation of general sharing mechanisms, and examples of large-scale Data Spaces. The key technologies and architectures for Industrial Data Spaces are also collected in [30] without focusing on actual implementations. In [31], a systematic survey focused on the architectural perspective of Data Spaces is introduced. Reference to Manufacturing Data Spaces exist; however, the survey is not focused on this domain.

In contrast, the present study adopts a distinct analytical approach by examining the literature from an application-oriented perspective, rather than concentrating on architectural considerations. In particular, the term *application* is used to refer to how something is used in a practical setting, and not as a software application. The *use case* term is used as well. It is more focused and describes a specific instance of use. Considering practical examples of Data Spaces in Manufacturing and following the methodology (Section 2), 26 works were identified and analyzed in the current study.

The paper is organized as follows. Following the Introduction, the methodology followed is analyzed in Section 2. The actual analysis of the works studied is available in Section 3. In Section 4, the findings of this work are discussed, and the conclusions are drawn in Section 5.

## 2 Methodology

### 2.1 Data collection and presentation

**Fig. 2:**
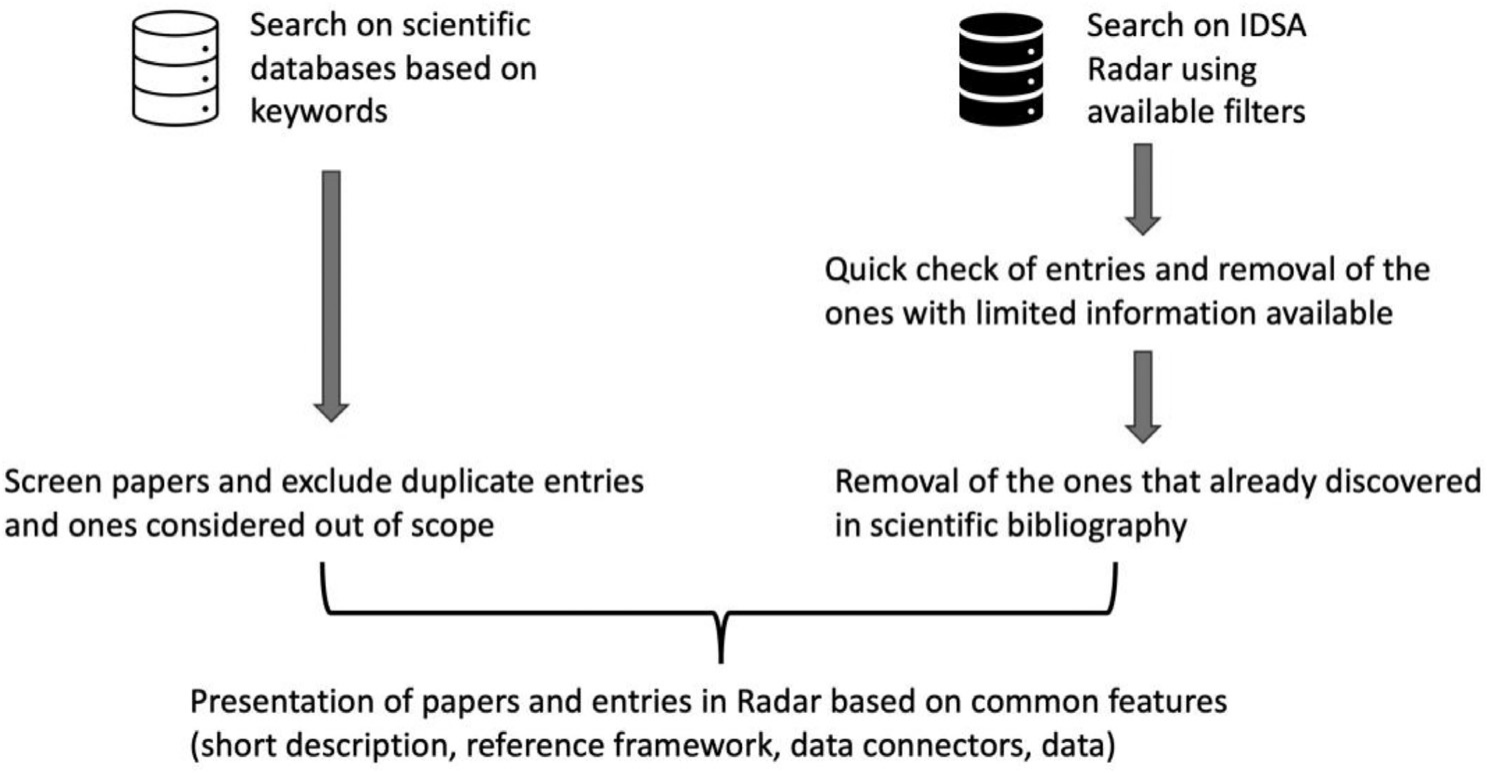
Methodology used for data collection and presentation

Data Spaces have attracted significant interest from both academia and industry. Therefore, in addition to scientific databases, the IDSA Radar [32] was considered during data collection to identify additional industry applications. The collection of works that was analyzed in this survey, was based on a multistep methodology (Fig. 2). The methodology followed was based on systematic review approaches [33] which in their first step include the search in scientific databases and online sources. After searching for information sources, the methodologies proceed with the processes for the selection or exclusion of articles based on defined criteria.

The most widely used scientific multidisciplinary databases, Google Scholar, Scopus, and Semantic Scholar were used to search for papers. The search criteria included articles from 2017 (Data Spaces concept became a trend in Europe, IDSA was established etc.) and onward. Articles published in journals, conference proceedings, and book chapters were considered. The main search keywords used were *“manufacturing data spaces”,* “*industrial data space*”, “*data spaces industry 4.0/5.0*”, etc. The results were filtered and only papers that included at least one use case related to Data Spaces implementations for the manufacturing domain were selected. Papers that presented concepts, architectures, etc. were removed because they were out of the scope of the current study.

**Fig. 3:**
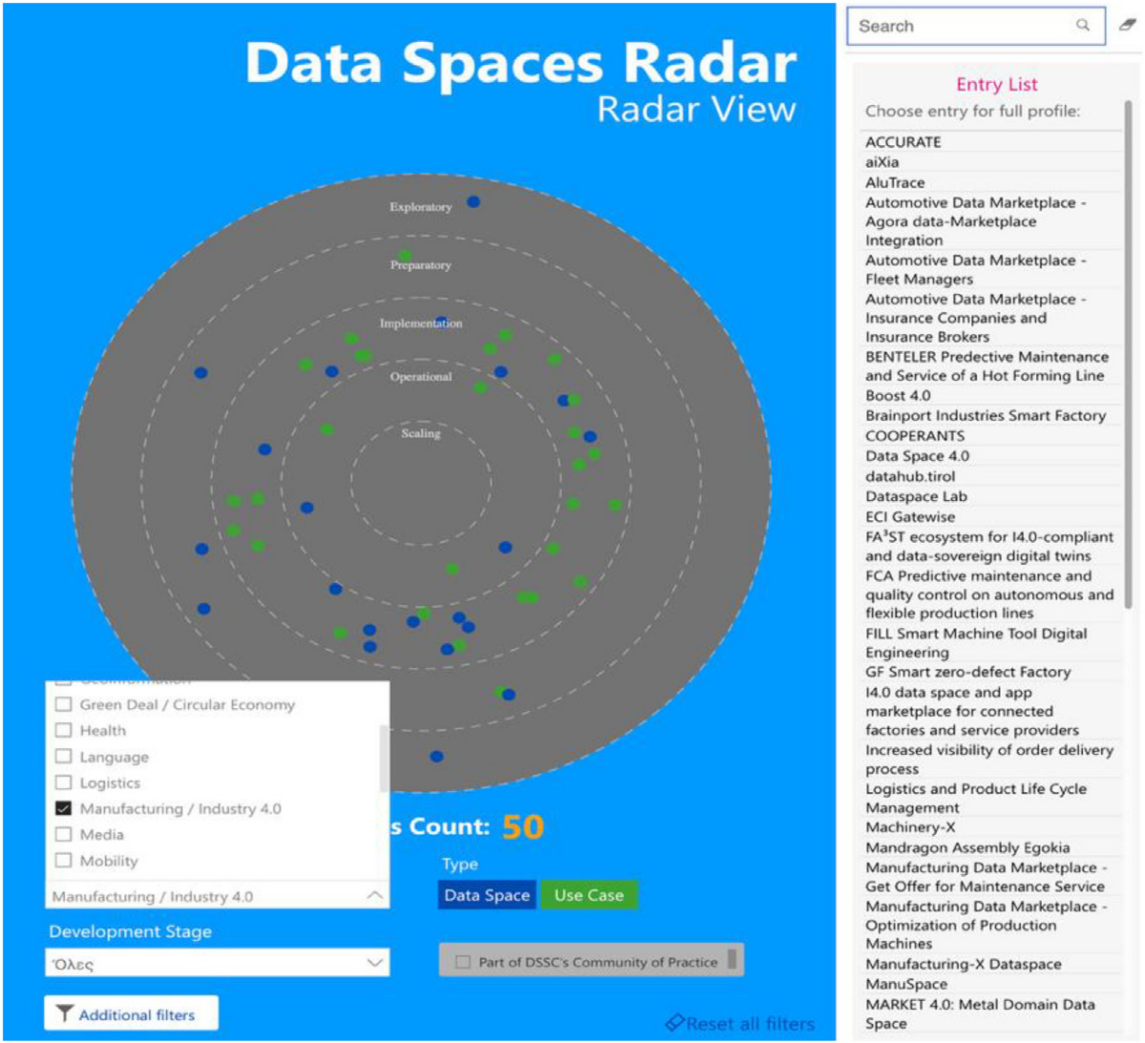
View of IDSA radar demonstrating manufacturing/industry 4.0 domain entries

As the Data Spaces are mainly business-related concepts and applications, many of them are not available in the current scientific bibliography. To address this gap, the authors supplemented their analysis by examining cases listed in the IDSA Radar (Fig. 3). It is an online tool that enables the searching for Data Spaces and provides filtering functionalities. It was considered as a source of information as it is aligned with DSSC guidelines and toolbox [34] (the Radar is part of the DSSC toolbox validation process). The Radar is hosted and maintained by IDSA with additional contributions from the DSSC community. It gathers information for all the well-known Reference Architectures (FIWARE, IDSA, DSBA, GAIA-X). The Radar [35] follows the DSSC terminology by providing mandatory fields during the registration process, with references to the DSSC glossary. For the purposes of the current study, the built-in filters of the Radar were used. In particular, the Manufacturing/Industry 4.0 domain filter was selected, and cases under this category appeared in the Radar. Before the analysis, non-EC entries were removed using the built-in filter for countries. After that, the available entries were quickly screened and the ones with limited information available were removed as no added value was provided by them. After removing any common (duplicate) Data Spaces applications’ references with the scientific bibliography, the rest are analyzed and presented.

To further enrich the content and the insights of the selected works, further research was conducted. Many entries contained references to supplementary documentation or related projects, which were systematically explored to extract additional insights. Regarding the Radar cases, further research was also conducted for the detected cases. Both the Internet and scientific databases were searched based on titles of detected Data Space cases, to discover connected resources that will enable the extraction of further information. This information was also screened and presented (in the cases needed) to provide a more coherent presentation of the works.

For all scientific papers and entries in the IDSA Radar, common information was extracted and presented (where available):

• a short description of Data Space application or use case
• the Data Space reference architecture was used
• the data connector implementation was used
• the development stage of each use case
• information regarding actual data used

This information was used in the Discussion section and informed the creation of the diagrams that presented there.

### 2.2 Limitations

Some limitations with respect to the current work and the respective methodology should be considered. Firstly, it should be noted that this work is not a Systematic Literature Review (SLR) regarding Data Spaces, but a shorter survey of available Data Spaces cases for Manufacturing and smart factories. Therefore, an SLR-based type of analysis regarding the number of papers, distributions in journals, conferences, etc. was not included.

Moreover, it should be clarified that this study is focused on Manufacturing domain (Table 1). However, in the eras of Industries 4.0 and 5.0, the Manufacturing domain is closely connected with other domains like Supply Chain and Logistics, Energy, etc. This fact affects the final decision on the inclusion or non-inclusion of the detected papers. Publications primarily focused on domains outside the manufacturing context were excluded. Nevertheless, papers that included cases related to other domains (e.g. Supply Chain) but having the optimization of production/assembly processes as center concepts (so they were closer to Manufacturing) were included. For example, the works that are related to servitization of Manufacturing and the discovery of suppliers usually consider the total value chain in a Manufacturing ecosystem including Supply Chain processes. So, these works were considered. Moreover, the IDSA Radar’s filters distinguish Manufacturing/Industry 4.0 from domains such as Automotive, Logistics, etc. So, relevant entries were not considered.

In addition, this work considers Manufacturing-related applications regarding Data Spaces in Europe. For entries retrieved from the IDSA Radar, geographic filtering was straightforward due to built-in functionalities. In contrast, in terms of bibliography, the process was not that straightforward. In the cases where there was no explicit reference to the geographic area of the application, other aspects were considered. They were authors’ affiliations and acknowledgments to any European or national research projects etc. For example, cases [30,36,37,38] were finally excluded as there was no reference to a foreseen application in Europe. The exclusion of these cases was further supported by the absence of any reference to known Data Space architectures coming from leading European initiatives. This can also be considered as a finding. Today, beyond Europe, the term Data Space is more connected to the definitions of distributed data storages and not to the aspects of data sovereignty and governance.

Finally, one should consider that a Data Space (Table 1 definition) does not define any minimum number of participants. Moreover, it is not required that participating entities originate from different organizations. Consequently, in this study, use cases that include Data Spaces concept and technologies for peer-to-peer communication between even just a couple of participants in both inter- and intra-company scenarios are considered. So, the study is not limited to use cases that include multi-stakeholder Data Spaces.

## 3 Manufacturing Data Spaces Applications

### 3.1 Manufacturing data spaces applications in bibliography

In 2022, a European Industrial Data Space (EIDS) framework [39], based on IDSA RAM, was introduced and covered use case examples of Data Spaces applications related to manufacturing within the Industry 4.0 framework. In particular, the EIDS principles were applied to cases related to predictive maintenance. In [40,41] a cognitive data analytics platform for the injection molding industry was introduced. The dataset consisted of time series data collected in real time from machines (current, pressure, etc.) and was available in JavaScript Object Notation (JSON) format. The platform implemented supervised and unsupervised learning techniques and utilized IDS Trusted Connectors [43] applied over the Message Queuing Telemetry Transport (MQTT) protocol. The analytics platform consumed data from the factory with injection molding machines, based on IDSA principles, to provide analytic services. The same platform was also used in [42] to build a Data Space use case for production scheduling optimization of a textile industry. The dataset was available as comma-separated value (CSV) files with information related to machine operation and production orders.

In [44] a machine monitoring use case based on EIDS principles for molding machines was proposed. The machine owner (factory) was considered the data provider and the analytics service provider the data consumer. In this case, time-series data, in JSON format, coming from sensors attached to machines were used. Similarly to previous cases connected to EIDS, the IDS Trusted Connector implementation was used.

Zero-Defect Manufacturing enabled by FIWARE-based Data Space concepts and the corresponding implemented connectors was introduced in [45]. The IDS Base Connector was extended by using FIWARE components such as context broker. Additionally, the IDSA RAM was considered. The implementation was based on the FIWARE for Data Spaces design principles [46]. Factory machines were the data providers. The data consumer was a predictive maintenance system for the milling machine and a quality control service evaluating the quality of production processes.

In [47], a Sustainable Manufacturing-as-a-Service (SMaaS) use case enabled by data space components is introduced. It was part of the Manufacturing-X initiative which is focused on the creation of secure and interoperable data interfaces, along with data-driven business models based on Gaia-X principles. In the experiment carried out, the Eclipse Data Space Connector (EDC) [48] was used to enable the sovereign and secure sharing of data in a marketplace. Using these connectors, a manufacturing company could share information such as manufacturing capacity and tasks in the marketplace, so a future customer can post a request to acquire as-a-service manufacturing. Providers of SMaaS were able to commit their offers using EDC as well.

Data Spaces technologies, serving as enablers of Manufacturing-as-a-Service (MaaS), were also examined in [49]. Within this context, facilitated the execution of remote production orders across the Data Space infrastructure. Asset Administration Shell (AAS) was used to model manufacturing assets and production orders. A use case that simulates a MaaS system where a customer and a supplier exchange production data according to standards was developed and tested. The use case considered the stamping and laser cutting processes based on customer orders. Manufacturing orders can be executed through an exchange between the IDS Connectors. Moreover, available assets and processes modeled using AAS are available through the IDS ecosystem. The Dataspace Connector was utilized for the scenario implementation.

MaaS Ecosystem for Catena-X [50] was another approach that connected the MaaS and Data Spaces concepts. Catena-X [51] is the first open and collaborative Data Space for the automotive industry, based on Gaia-X. Catena-X offers a series of use cases including demand and capacity management, digital twins, traceability, modular production, and recently MaaS. MaaS for Catena-X provides services for information extraction and integration, manufacturing capabilities representation, supplier search, and connection to external marketplaces. In [50], the MaaS Catena-X use case scenario included a Portal for supply chain visualization, EDC connectors for sovereign data exchange and interoperability with the Catena-X ecosystem, a Smart Factory Web (SFW) [52] connector for data mapping and transformation, and search services. By combining these components, a prototype of a federated MaaS marketplace was introduced. JSON and Computer-Aided Design (CAD) files were supported for the representation of manufacturing assets.

Umati Data Space [53] introduced new business models, governance and security services for the company. It enabled selling a machine service instead of the machine, as Data Spaces technologies enabled the machine builder to connect to the machines of its customers and provide a data-driven maintenance platform. The implemented use case was a data hub that included a community of components, machine manufacturers and operators. The use case demonstration included a 365 × 24 × 7 testbed for all interested machine builders. This as-a-service case was based on IDSA and Gaia-X architectures.

In [54], the Data Space concept was used as an enabler for a Federated Learning (FL) scenario for collaborative monitoring, predictive diagnostics, and maintenance. The federated Data Space was established based on IDSA and Gaia-X principles. Core Gaia-X services such as Broker, Data Catalogue, and Identity Provider were parts of the federated cloud ecosystem. Connectivity within the ecosystem was facilitated through the deployment of data connectors by each participating entity. Every FL local module was part of the data connector. So, the local models become available to cloud global model by using connectors and the Gaia-X based federated ecosystem. The authors did not provide information on the connector that was used. An open predictive maintenance dataset consisted of 100 machines and included machine’s characteristics, situation telemetry per hour, and failures was used.

A Data Space approach for Industrial Roll-to-Roll Label Printing Manufacturing was proposed [55]. The Data Space infrastructure was installed at the authors’ university premises and was mainly a persistence database. Besides the data management functionalities, the Data Space platform supported data analytics, MQTT connectivity, visualizations, semantic context brokering, security and governance. It was used to enable an error handling and detection solution for the printing industry. The data were error-related information associated with printing operations in RDF [56], JSON and JSON-LD [57] formats. The authors did not mention any reference to architectures such as IDSA, Gaia-X etc. however they considered interoperability, security, and governance aspects that are key concepts of Data Spaces.

FLEX4RES, an open framework for resilient manufacturing based on

Data Spaces was introduced [58]. The case study was related to a Learning Factory. The framework was applied for unexpected events such as disrupted supply chains, machine downtime, etc. The data exchange and sharing processes were conducted within a Data Space based on IDS and Gaia-X components. IDS connector was considered for data exchange over the IDS and Gaia-X infrastructure. Resilient assessment services were provided by the FLEX4RES framework based on system theory, anomaly detection, and self-orchestration. A consumer could search for services in the Gaia-X Federated Catalogue.

A Data Space approach for a Digital Twin framework aiming at the representation of machines in a shopfloor is proposed in [59]. The framework was based on AAS for machine and asset modeling. Data security and sovereignty were enabled by using Data Spaces components. IDS reference architecture was followed for the implementation. The implemented case study was based on the Dataspace Connector [60] to enable the transfer of data coming from machines, sensors, etc. In addition, a DataSpaceApp4EDI application was included. The application orchestrated the Dataspace Connector’s actions, managing data provision and consumption. IDS Broker, Identity Provider, and Clearing House were also mentioned; however, there are no details on their implementation. The use case included two companies, from Spain and Portugal, that were able to track the status of the machines over time and monitor the cycle time of production based on the Data Spaces information and implemented Digital Twins. Additionally, companies could use a Machine Configurator App to simulate the machine purchasing process and evaluate the available machines so to select the best suitable one that met the company’s requirements.

Another Digital Twin application enabled by Data Spaces was introduced [61]. It was designed to allow prediction of machine lifetime for cutting tools. It was based on an FL approach, and the modeling was enabled by AAS. The data exchange between global and local nodes/agents of the FL approach was enabled by a shared Data Space based on Gaia-X architecture. The use case enabled intelligent analysis and decision-making under the concepts of data sovereignty and transparency. With respect to the connectors, the EDC implementation was utilized . Data used for the realization of the scenario included machine real time series data (current, speed, etc.) and Manufacturing Execution System (MES) metadata.

In [62], the concept of Data Spaces was explored as a novel data management paradigm; however, the study did not emphasize data sovereignty, which is a fundamental characteristic of Data Spaces within the context of the manufacturing multi-value chain. In this case, there are no reference in architectures like IDSA and Gaia-X and the Data Space was mainly a collection of all subject-related data and their relationships. Data in this Data Space were collections of many heterogeneous sources and relationships generated by the manufacturing companies in collaborative value chains. An example in the electric manufacturing industry was presented along with experiments in the Brunel University London Advanced Manufacturing Laboratory.

In [63], a data sharing ecosystem for the production sector was proposed, including suppliers and manufacturers. It was established to support Digital Product Passport (DPP) realization as each ecosystem participant contributes his/her data to the DPP, creating a traceable aggregation of product information. The ecosystem was a Data Space following Gaia-X and IDSA architecture principles. The production data used were energy consumption and data from MES or Enterprise Resource Planning (ERP) systems. Events related to these data were modeled and associated with product IDs as part of the DPP information. The events were stored and accessed through the Data Space. The scenario is still under development. EDC connectors are planned to be used.

Smart Factory Web (SFW) [64] provided interoperability and integration with AAS to enable companies to publish factory capabilities and assets’ details on manufacturing marketplaces [65]. Its main contribution was the connection of standards, such as IDS, AAS and OPC UA. A use case for collaborative condition monitoring of factory assets was presented. The OPC UA Factory Connector implementation was introduced to enable the creation of SFW. The connector acted as a gateway among SFW participants and customers.

An Ontology-based Data Space [66] for battery production was proposed. The implemented study included a Li-ion battery cell production facility and was a lab-scale experiment. The Data Space that was created, covered data from the various stages of battery cell production and it was a collection of databases and triple stores. Domain ontologies related to battery production and materials were used. Gaia-X architecture was mentioned without any reference to data connectors. Querying functionalities of this graph-based Data Space were based on SPARQL [67]. AI algorithms were applied for defect detection and quality inspection over the Data Space. Pictures of electrodes and their tests were the data used.

### 3.2 Manufacturing data spaces applications reported in the IDSA radar

A Data Space related to additive manufacturing was implemented based on IDS connectors and Broker. The latter acted as a marketplace that enabled data in the Material Data Space (AIuTrace) [68] to be findable. The available data enabled the creation of an algorithm that offers an optimized design. The materials were additive-made aluminum parts based on laser powder, and the objective of the algorithm was to support designers. Both material processes and parts were semantically modeled. There is no reference regarding the connectors’ implementation used.

FA3ST ecosystem for Industry 4.0 [69] was based on the combination of AAS with IDS to create digital twins that supported data sovereignty. Both product and production data were represented by AAS and was shared across the company using IDS connectors (EDC implementation). By combining IDS with AAS, interoperability and sovereignty were ensured.

I4.0 Data Space and marketplace [32] for connected factories and services were implemented as a prototype. App Store and Connectors were used based on IDSA RAM. Connectors were used by a factory that acts as a data provider. The App Store enabled the discovery of predictive maintenance providers that consumed the data through the connectors (Dataspace implementation was used).

OSME case [70] related to the connection of an OEM manufacturer and its suppliers, aimed to increase visibility of order delivery processes. It targeted the establishment of a reliable and seamless data flow between the manufacturer and suppliers. The case included the existing infrastructure of the companies and the usage of IDSA RAM components. The suppliers were able to access only the needed data and no further production data that stayed with the manufacturer. There is no reference regarding the connectors that were used.

EGOKIA platform [71] based on IDSA connectors facilitated the implementation of a use case involving the integration of data from a manufacturer’s assembly line with AI platforms deployed across distributed plants. This enabled collaborative learning and knowledge sharing. The Data Space promoted trusted access within the participants in EGOKIA and enabled the creation of new business models based on FL. There is no information about connectors’ implementation.

ManuSpace for pharmaceutical manufacturing [72] promoted trusted data sharing by enabling secure data exchange between the manufacturing company and the sponsor pharmaceutical company. It promoted the permission-based sharing of maintenance and plant location data to a contract company to provide relevant services. ManuSpace was connected to a marketplace to enable the sharing of quality and environmental data through the Data Space that is in a preparatory stage, and its implementation is based on FIWARE.

Market4.0 ecosystem [73] was reported to enable a series of Data Space-related cases. IDSA RAM was mentioned; however, no detail of the actual implementations was provided. 3DFORM platform, ENTER experiment, and Metal Domain Data Space are cases related to Data Spaces in Market 4.0 based on the same IDSA Radar entry [73]). 3DFORM platform offered data sovereignty and control between the cloud platform and service users in the Market 4.0. ENTER enabled the connection of production equipment suppliers for metal processing. An ordering app, a visualization app, and an equipment selection service enabled the scenario realization. Similar is the case of Metal Domain Data Space that linked inventories of different equipment manufactures based on IDSA RAM. A Plastic Industry Data Space is also mentioned without including many details.

A Milk Industry Data Space use case was reported as part of knowlEdge compute continuum [77] that promotes AI-powered manufacturing. Modern milk industries are considered part of the Manufacturing domain in the Industries 4.0/5.0 eras as they include production processes based on automation, IoT, and AI. The Dataspace Connector implementation and IDSA RAM were used to enable sovereign data transfer related to production orders of a milk producer. The data consumed by knowlEdge Decision Support System to provide analytic services related to demand forecasting and production scheduling optimization [74].

Pressious Data Space, for offset printing manufacturing, was implemented based on IDSA principles. It was part of open experimentation in the AIREGIO project [78]. The offset printing company shared historical measurements from a 4-month period (machine data, orders, and waste management data) with research partners to provide machine learning services for production monitoring and waste management [75]. Dataspace Connectors was used for the establishment of the Data Space.

The Smart Connected Supplier Network (SCSN) [76] enabled manufacturing companies to exchange order-related data. Companies must register once with an SCSN Service Provider so to exchange data (orders, invoices, technical product data, etc.) with all other affiliated companies in the production chain. The data in SCSN were described by an XML schema and companies determined which data was shared. SCSN improved supply chain productivity through fast, secure and interoperable information exchange based on IDSA RAM.

## 4 Discussion

The main findings of this study are related to the application areas of Data Spaces in Manufacturing, the reference architectures and actual connectors used based on the presentation of the works in Section 3. It should be noted that all the results, numbers, and figures presented in this section are related to the sample of 26 cases studied in the previous section and the information was extracted from them.

**Fig. 4:**
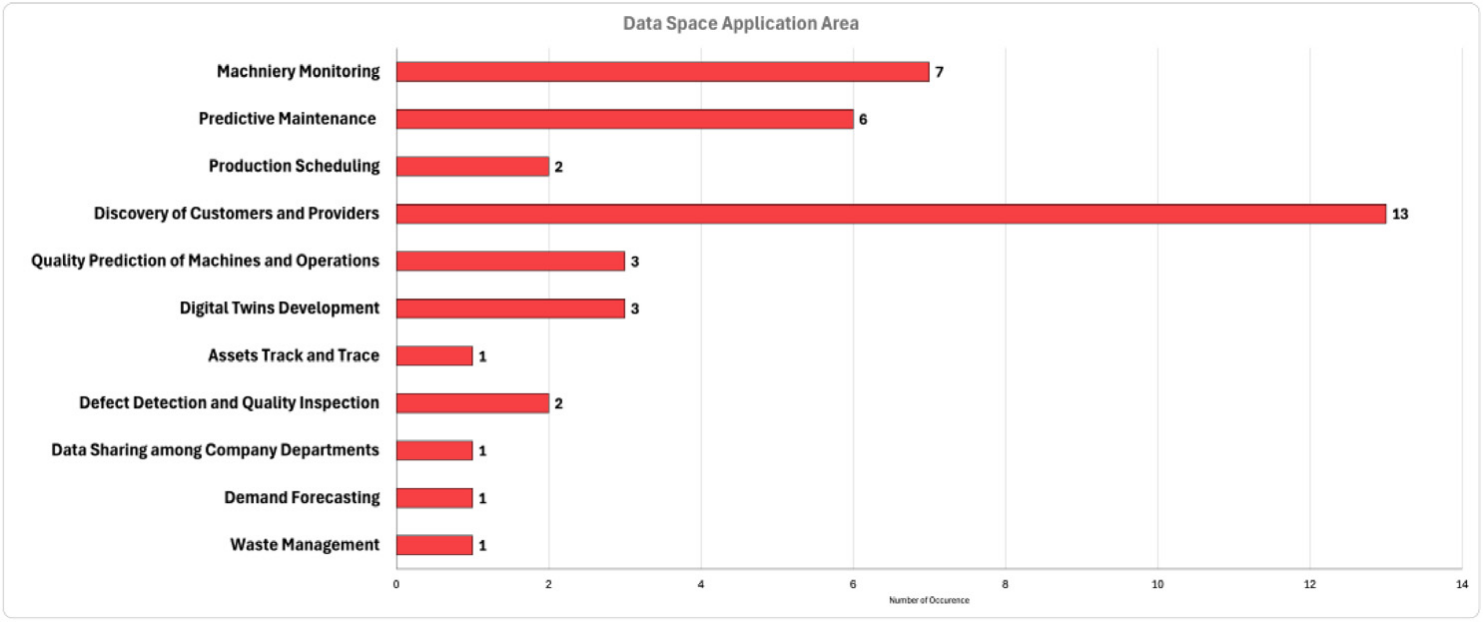
Manufacturing Data Spaces Application Areas

The applications of the Data Spaces for Manufacturing were categorized by the authors in high-level application areas, as shown in Fig. 4. Data Spaces technologies are used mainly in collaboration scenarios that require the discovery of customers/providers and were mentioned in 50% of the studied works. It should be highlighted that the concept of Data Spaces is already adopted in state-of-the-art approaches [47,49,50] for collaborative manufacturing, such as the newly introduced concept of MaaS. Predictive maintenance was detected as the second most common application area, with a percentage of 23%. However, it should be noted that applications strictly related to predictive maintenance, such as machine monitoring and quality prediction were reported in more than 60% of the total cases. Therefore, almost 2 out of 3 cases identified in this study were related to Data Spaces applications for predictive maintenance and corresponding activities.

In addition to Fig. 4 findings, the sustainability aspect can also be considered in some of the applications based on the analysis of Chapter 3. In [63] the realization of a Digital Product Passport application, and in [47] a Sustainable MaaS use case were enabled by Data Space components. In addition, the zero-defect manufacturing application [45] enabled by FIWARE-based Data Space connectors is also related to sustainability [79,80]. These findings related to sustainability can provide further justification for recent works [81] that present Data Spaces as sustainability enablers.

**Fig. 5:**
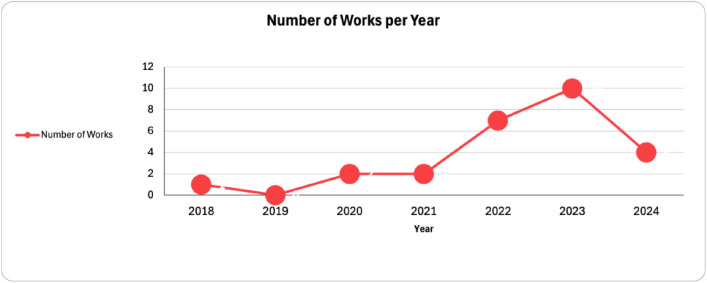
Number of Works Related to Manufacturing Data Space Applications per Year

Another finding is the increasing trend in Data Spaces applications in Manufacturing domain. As depicted in Fig. 5, more than 80% of the cases are reported from 2022 and onward (less than 20% from 2018 to 2021). It can be considered that the concept and technologies of Data Spaces have matured more in the last couple of years, making their adoption easier. The European Data Strategy [3] published in 2020 can also be considered as a key factor for this increase, as the impact of the strategy has started to emerge a couple of years later. It also supported IDSA, Gaia-X and FIWARE to evolve. Furthermore, this rising trend could also mean that companies and developers are now more familiar with the Data Spaces concept and that this is more accepted. A combination of the increased maturity of the Data Space concept, the impact of European Data Strategy and the acceptance of stakeholders could also be the key factor for this increase.

**Fig. 6:**
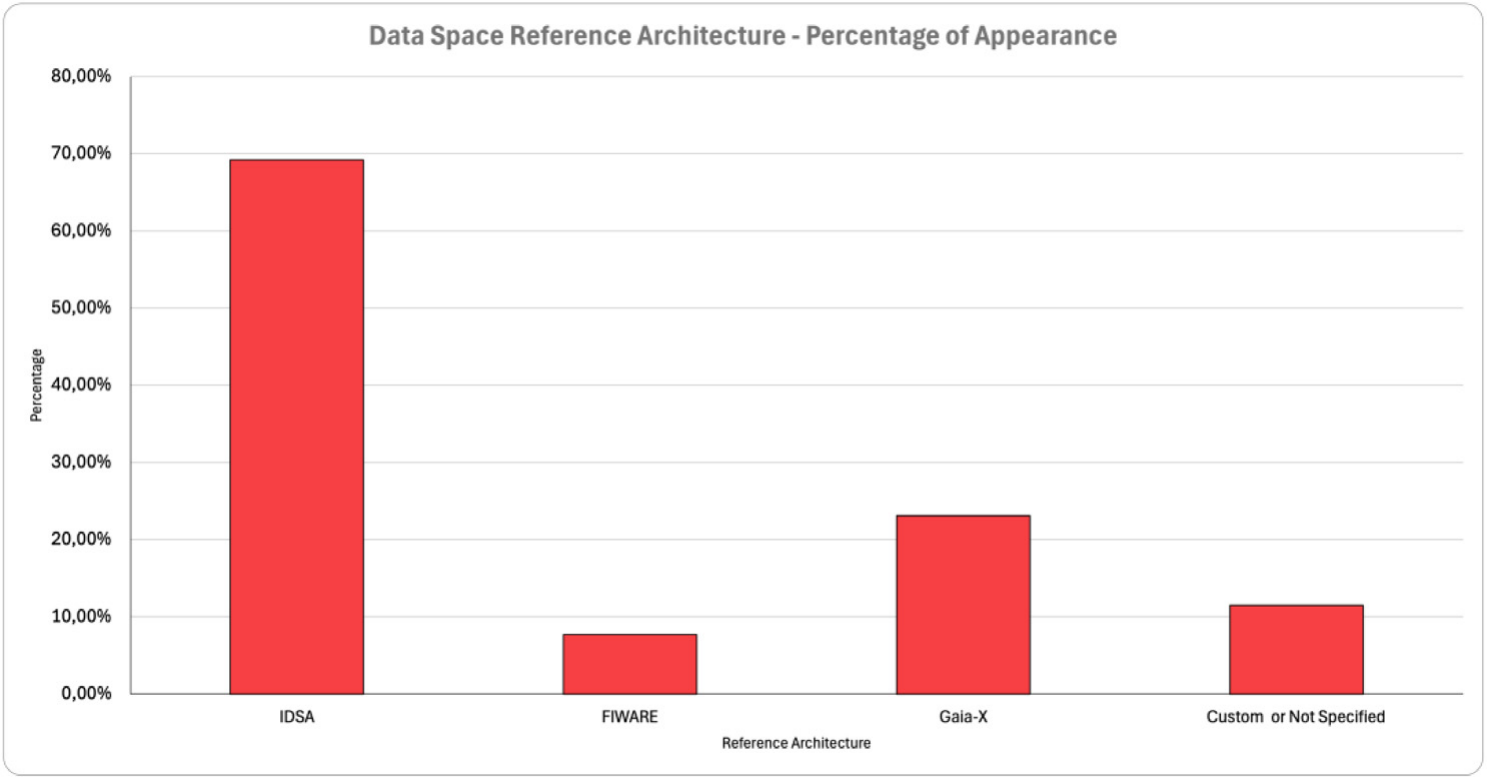
Percentage of Appearance from Various Data Spaces Related Architectures in Manufacturing Domain

The Data Space-related efforts in Europe are led by a series of reference architectures and corresponding initiatives. This information was extracted from the studied works and is presented in Fig. 6. IDSA seems to be the reference architecture that drives the developments regarding Manufacturing Data Spaces, as it appeared in more than 69% of the cases. Gaia-X was the second most used architecture (over 23%). This is considered as expected as Gaia-X is newer than the IDSA, and it is focused on cloud infrastructure and not only to Data Spaces as IDSA. In many cases, the two architectures are complementary. An interesting finding is that in 12% of the cases, there is no reference to any of the known architectures. This means that the adoption of reference architectures for Data Spaces is not universally followed by implementers. The usage of no ’standardized’ reference architectures may significantly decrease the interoperability in large-scale Data Spaces, but this cannot be clearly extracted by the findings of the current study.

**Fig. 7:**
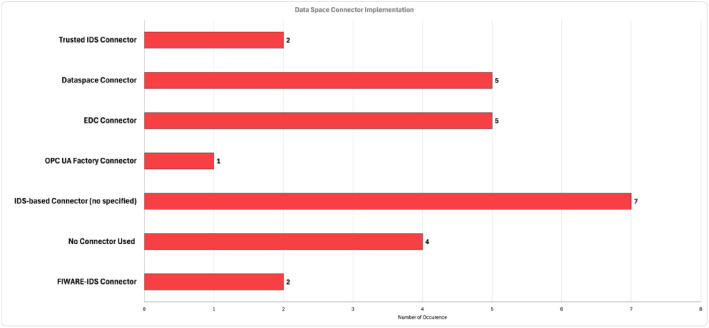
Number of Appearance from Various Data Space Connector Implementations in Manufacturing Domain

The key technological enabler for setting up a Data Space is the data connector. In Fig. 7, a categorization of the different implementations of Data Space connectors based on the studied sample is presented with respect to the Manufacturing domain. It is depicted that the Fraunhofer Dataspace Connector and Eclipse Foundation EDC Connector are the most often used connectors. An interesting finding could be considered that in more than 15% of the studied works there is no reference to a data connector implementation connected to any of the reference architectures. Moreover, in almost 27% of the studied works, just an IDS-based Connector is highlighted with no reference to any specific implementation. The latter could lead to thoughts for further ’custom’ implementations to be available, or to thoughts regarding the maturity level of Data Space-related applications. Another finding is the low percentage of FIWARE-IDS connector, about 8%. This result can be expected, as the FIWARE concept is mainly adopted in Smart Cities and Mobility domains.

**Fig. 8:**
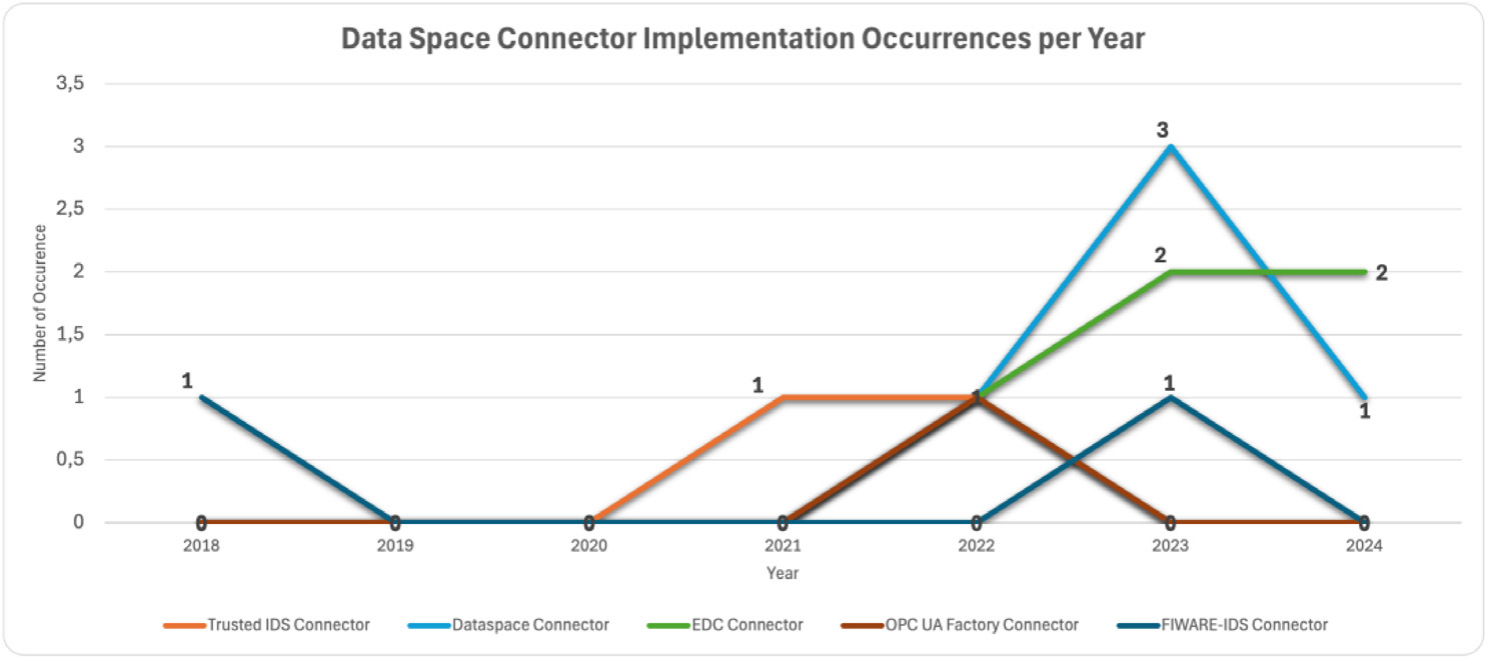
Number of Data Space Connectors Implementation per Year regarding Manufacturing Domain

In Fig. 8 the occurrences of various implementations throughout the years are presented. However, the extraction of valuable information is limited by the small number of available cases. The increasing trend of the EDC connector can be considered as a finding. Also, the Dataspace Connector is not maintained anymore, and this is visible in its decreased appearance in 2024.

**Fig. 9:**
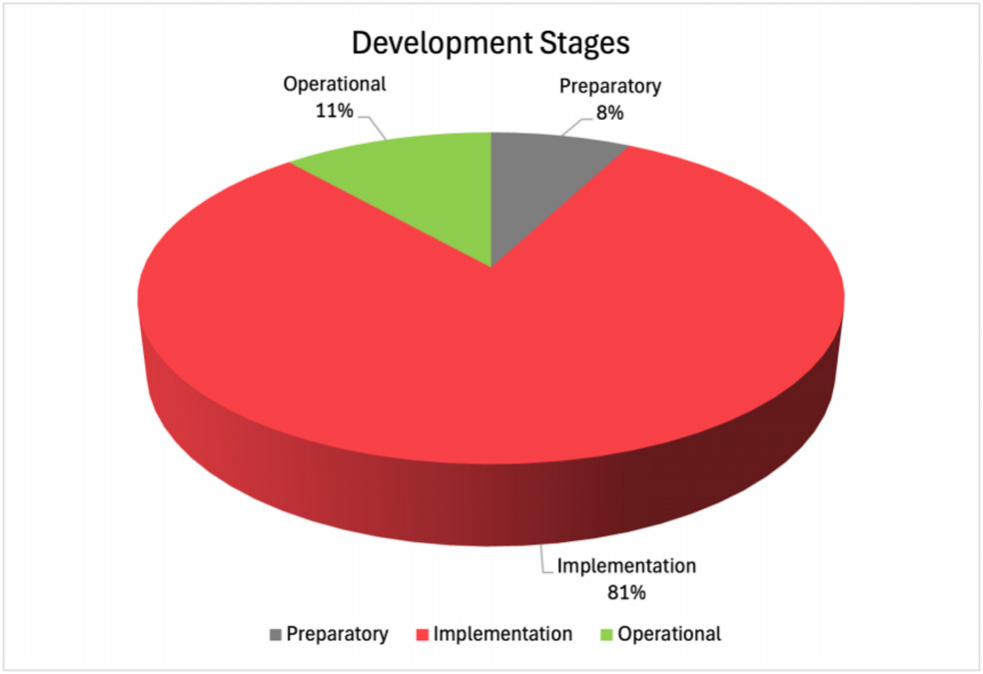
Development Stages of Manufacturing Data Spaces Applications

The development stage of the Manufacturing Data Spaces applications detected is also listed in Table 2. DSSC terminology was followed for the characterization of the development stages [82]. Therefore, five development stages were considered: Exploratory, Preparatory, Implementation, Operational, Scaling. For the cases available in the Radar, the characterization given by the reporters of the Data Spaces applications was adopted. For the rest of the cases, it was extracted by the authors, who analyzed the articles and mapped their stage to the five development stages. It should be noted that all the information is coming from the sources studied. Some of the cases may have been evolved after their documentation. In Fig. 9, the percentages of each development stage for the sample studied are presented. Most of the cases studied (81%) are in the implementation stage. This stage typically involves a Data Space pilot. Therefore, this high number indicates that most of the cases studied were tested in pilot settings, but they are not operational yet. This finding can relate to the current EC-funded research landscape. As mentioned in the Introduction, EC-funded projects like UNDERPIN and SM4RTENANCE for the deployment of Manufacturing Data Spaces are in progress. In addition, most of the studied cases come from research projects, so their goal is the pilot application of the Data Spaces and not to reach the operational stage (11% of the cases). Another finding can be considered the absence of Scaling stage regarding the Manufacturing domain. This may be justified by the fact that in the current study, only Manufacturing related cases were considered from the large operational Data Spaces, Catena-X and SCSN.

**Table 2:** Summary and key aspects of the presented works.

Data Space Name and Reference	Data Space Application(s)	Data Space Technologies and Concepts	Development Stage
Cognitive Analytics Platform for Industry 4.0 based on EIDS [41,42] (2022)	Machinery Monitoring, Predictive maintenance, production scheduling	IDSA RAM, IDS Trusted Connector	Implementation
Predictive Maintenance Platform based on EIDS [44] (2021)	Predictive maintenance	IDSA RAM, IDS Trusted Connector	Implementation
Zero-Defect Manufacturing enabled by FIWARE-based Data Space [45] (2018)	Predictive maintenance, defect detection and quality inspection	IDSA-RAM, FIWARE, FIWARE-IDS Connector	Implementation
Manufacturing-X Sustainable Manufacturing-as-a-Service (SMaaS) [47] (2023)	Discovery of customers and providers	EDC Connector	Implementation
Manufacturing-as-a-Service (MaaS) enabled by Data Space components [49] (2022)	Discovery of customers and providers	Dataspace Connector, IDSA RAM	Implementation
Manufacturing-as-a-Service Ecosystem for Catena-X [50] (2023)	Discovery of customers and providers	EDC Connector, Gaia-X	Operational
Umati Data Space Story [53] (2022)	Machinery monitoring, predictive maintenance	IDSA RAM. Gaia-X	Operational
Federated Learning based on Data Spaces concept [54] (2023)	Machinery monitoring, predictive maintenance	IDSA RAM, Gaia-X Broker, Catalogue and Identity provider	Implementation
Data Space for Industrial Roll-to-Roll Label Printing Manufacturing [55] (2023)	Quality prediction of machines and operations	No info available	Implementation
FLEX4RES framework for resilient manufacturing [58] (2023)	Quality prediction of machines and operations, Discovery of customers and providers	IDS Connectors (no specific implementation), Gaia-X Service Catalogue	Implementation
Data Space approach for Digital Twin [59] (2023)	Machinery monitoring, Quality prediction of machines and operations, Digital Twin	Dataspace Connector, IDS Broker, Identity Provider and Clearing House	Implementation
Digital Twin Data Space based approach [61] (2024)	Machinery monitoring, Digital Twin	Gaia-X. EDC Connector	Implementation
Manufacturing Multi- value Chain Collaborative Data Space [62] (2022)	Discovery of customers and providers	No info available	Implementation
Digital Product Passport based on Data Spaces [63] (2024)	Products track and trace	IDSA RAM and Gaia-X, EDC Connector	Preparatory
Ontology-based Data Space for Battery Production [66] (2024)	Defect detection and quality inspection	Gaia-X	Implementation
Smart Factory Web [64] (2022)	Discovery of customers and providers, Machinery monitoring	IDSA RAM, OPC UA Factory Connector	Implementation
AIuTrace Material Data Space [68] (2022)	Discovery of customers and providers	IDS Connectors (no specific implementation)	Implementation
FA3ST ecosystem for Industry 4.0 [69] (2022)	Discovery of customers and providers, Digital Twin	IDSA RAM, EDC Connector	Implementation
I4.0 Data Space and Marketplace [32] (2020)	Discovery of customers and providers	IDSA RAM, Dataspace Connector	Implementation
OSME Showcase: Increased Visibility of Order Delivery Process [70] (2024)	Discovery of customers and providers	IDSA RAM	Implementation
EGOKIA for Distributed Plants Connection [71] (2023)	Data sharing among departments	No info available	Implementation
ManuSpace for Pharmaceutical Manufacturing [72] (2023)	Discovery of customers and providers	FIWARE, FIWARE-IDS Connector	Preparatory
Market4.0 Ecosystem [73] (2021)	Discovery of customers and providers	IDSA RAM	Implementation
Milk Industry Scheduling Optimization supported by Data Spaces concept [74] (2023)	Demand forecasting, production scheduling	IDSA RAM, Dataspace Connector	Implementation
Pressious Data Space Approach for Offset Printing [75] (2023)	Machinery monitoring, predictive maintenance, waste management	IDSA RAM, Dataspace Connector, Broker	Implementation
Dutch Smart Connected Supplier Network (SCSN) [76] (2020)	Discovery of customers and providers	IDSA RAM	Operational

Regarding the data used in the Data Spaces related to the Manufacturing domain, there was high heterogeneity in the level of detail that each of the studied works was provided, and in many cases, the data-related information was limited. This limited information may also relate to the absence of components such as the IDS Broker and Gaia-X Catalog in more than 85% of the cases (optional components of a Data Space). However, they are important as they describe data and metadata connected to a data source. Therefore, any kind of detailed categorization and presentation of findings in a structured representation was difficult be- cause in many cases, there were limited references regarding the data and data formats. The issue of heterogeneous data available in Data Spaces is continuously gaining interest in the research community [83]. The need for data and metadata description in Data Spaces such as Manufacturing was defined by the authors [84], who introduced a Metadata Broker capable of covering specific aspects of manufacturing data and metadata.

In Table 3, the available data and corresponding formats for each application area (as defined in Table 2) are collected and categorized. The table includes high-level information per application area. Data format information was not available in some of the studied works. Machine and asset data are mainly available in the studied sample. The semantic representation based on RDF was detected as a key element for the description of data related to Manufacturing Data Spaces. The most popular data formats were JSON (the most popular), JSON-LD and CSV.

**Table 3:** Data and data formats in defined data space application areas.

Data Space Application Area	Data	Data Format
Machinery Monitoring	machine data, sensor data, asset data	JSON
Predictive Maintenance	machine data, sensor data	JSON, CSV
Production Scheduling	machine data, orders, sales data, assembly data	CSV
Discovery of Customers and Providers	asset data, orders, offers, capabilities	JSON, XML, CAD
Quality Prediction of Machines and Operations	machine data, asset data	RDF, JSON, JSON-LD
Digital Twins Development	machine data, asset data	RDF, JSON, JSON-LD
Assets Track and Trace	asset data, orders, location data	JSON
Defect Detection and Quality Inspection	asset data, multimedia	RDF/OWL, JPG
Data Sharing among Company Departments	assembly data	-
Demand Forecasting	sales data	-
Waste Management	machine data, environmental data	-

**Fig. 10:**
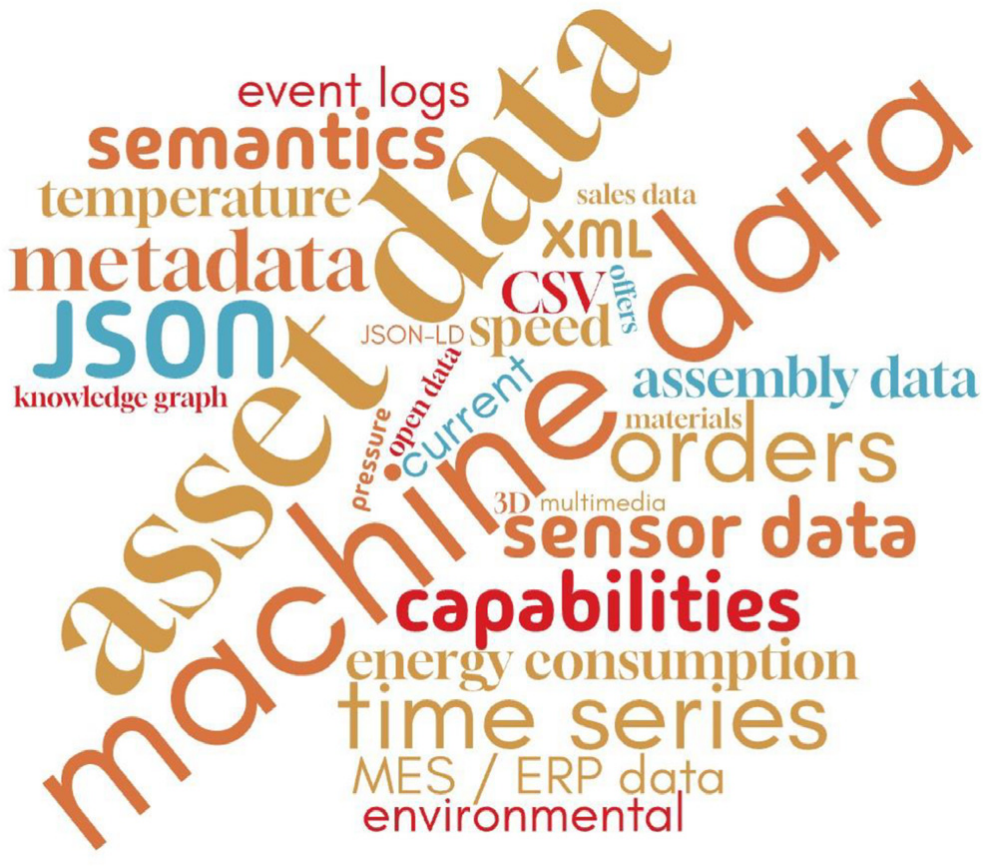
Word Cloud regarding Data and Data Formats in Manufacturing Data Spaces Applications

To account for the lack of information and the heterogeneous level of detail, a word cloud figure (Fig. 10) was created. This kind of visualization is considered for the cases in which precise and quantitative insights cannot be presented. The font size of every entry in the word cloud represents its appearance in the studied sample. Moreover, in a word cloud visualization it is easier to combine different data types. Fig. 10 was able to capture information about data type, data sources, data formats and representation.

Machine data (mainly time series data) were the most reported. Data related to orders, assets/products and manufacturing capabilities were also highly used. These findings are aligned with those related to the application areas of Data Spaces in Manufacturing. Machine data is the cornerstone for building predictive maintenance and monitoring services. To build collaborative manufacturing networks, data about products and offers are needed. Considering the rising concept of MaaS, the representation of manufacturing capabilities is a core idea that enables resilient manufacturing ecosystems.

**Fig. 11:**
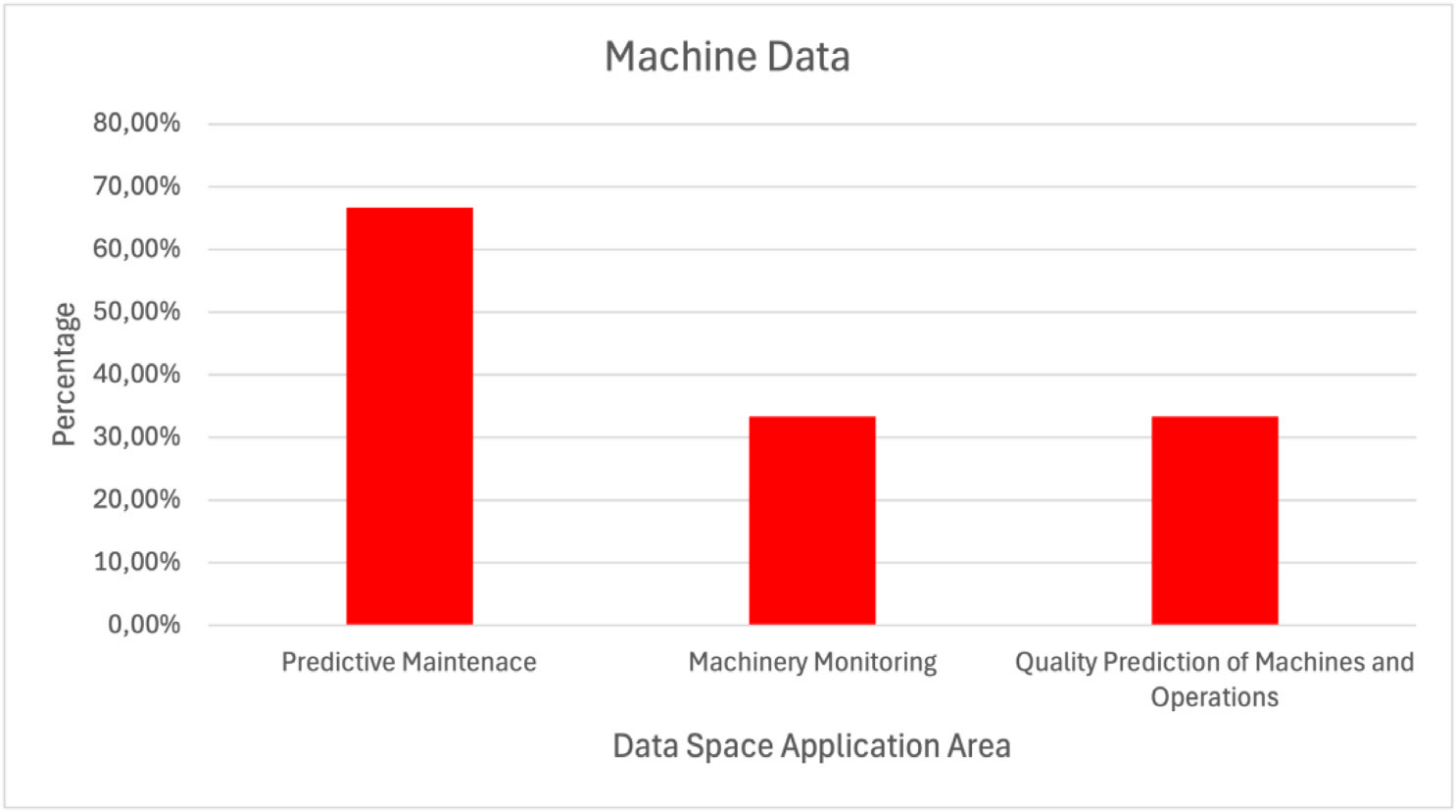
Machine Data Used in Various Manufacturing Data Space Application Areas

**Fig. 12:**
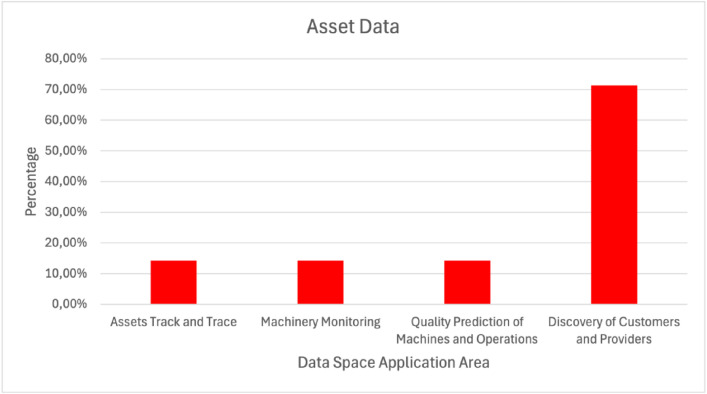
Asset Data Used in Various Manufacturing Data Space Application Areas

Regarding the two most used data types, machine and asset data, some additional findings were exported. Machine data (Fig. 11) were used in almost 70% of the cases that were related to predictive maintenance. Regarding asset data (Fig. 12), they used in more than 70% of the cases related to the discovery of customers and providers.

## 5 Conclusions

An analysis of Data Spaces applications in the European Manufacturing domain is presented in this paper and useful insights are extracted. Foremost, it was highlighted that there are only a few large-scale Data Spaces with most of the reported cases to be applications based on Data Spaces concept. The reported applications aimed to connect data providers with data consumers, enabling the latter to use the received data to deliver specific services. Moreover, high heterogeneity in reference architectures and Data Space connectors was identified. Furthermore, the absence of reference architectures usage or the non-adoption of well-known data connectors were detected. The heterogeneity issue was also highlighted in data types and formats.

Predictive maintenance and customer discovery applications were reported as the most widely used. Assumptions about architectures and data connectors that will prevail in the domain can be made. IDSA and Gaia-X are the main architectures used, and this seems to be the case for the future as well along with the rise of the EDC Connector.

Regarding the future directions of the current study, they include the addition of domains connected to Manufacturing such as Automotive, Logistics etc. Considering the rise of the MaaS concept, Manufacturing Data Spaces will include stakeholders’ data coming from the supply chain, so to enable collaborative and resilient manufacturing. Moreover, the search methodology can be extended considering information from relevant research projects. Many funded projects, especially under EC Cluster 4 for Digital, Industry and Space [85], are developing Data Spaces related to Manufacturing, so new results will be available. The Manufacturing Data Spaces’ contribution to sustainability and circular economy is also a topic which attracts interest, and an analysis of such aspects would shed light in the contribution of Data Spaces to the United Nations Sustainable Development Goals [86].

In addition, a further analysis of the connected and federated Data Spaces concepts can be conducted. Key elements of these concepts should be extracted and aligned with current implementations regarding Data Spaces for Manufacturing, to enable and promote the creation of Common European Data Spaces for this domain.

## Credit Author Statement

**Alexandros Nizamis:** Conceptualization, Methodology, Validation, Investigation, Resources, Data curation, Writing- Original draft preparation, Writing - Review & Editing, Visualization; **Panagiotis Gkonis:** Writing - Review & Editing, Supervision; **Dimosthenis Ioannidis:** Writing - Review & Editing, Supervision; **Aristotelis Ntafalias:** Conceptualization, Writing - Review & Editing; **Dimitrios Tzovaras:** Writing - Review & Editing, Supervision; **Panagiotis Trakadas:** Writing - Review & Editing, Supervision.

Data:This a review paper so there is no data associated with it.

## Data Availability

Review Data (Reference data).
